# Evaluation of Risk Factors for Peste des Petits Ruminants Virus in Sheep and Goats at the Wildlife-Livestock Interface in Punjab Province, Pakistan

**DOI:** 10.1155/2016/7826245

**Published:** 2016-05-15

**Authors:** Aziz-ul-Rahman, Muhammad Abubakar, Muhammad Hidayat Rasool, Shumaila Manzoor, Muhammad Saqalein, Muhammad Rizwan, Muhammad Munir, Qurban Ali, Jonas Johansson Wensman

**Affiliations:** ^1^Department of Microbiology, Government College University, Allama Iqbal Road, Faisalabad 38000, Pakistan; ^2^National Veterinary Laboratory, Park Road, Islamabad 44000, Pakistan; ^3^Progressive Control of Peste des Petits Ruminants (PPR) in Pakistan, FAO, Islamabad 44000, Pakistan; ^4^The Jallo Wildlife Park, Lahore 54000, Pakistan; ^5^The Pirbright Institute, Ash Road, Pirbright, Surrey GU24 0NF, UK; ^6^Department of Clinical Sciences, Swedish University of Agricultural Sciences, P.O. Box 7054, 750 07 Uppsala, Sweden

## Abstract

Peste des petits ruminants virus (PPRV) is causing infectious disease with high morbidity and mortality rate in domestic and wild small ruminants of Pakistan with valuable economical losses. The present study was carried out to investigate risk factors of PPRV in domestic small ruminants which were present in the vicinity of wildlife parks. A total of 265 sera samples (27 wild ruminants and 238 domesticated small ruminants) from apparently healthy animals from two different wildlife parks were collected and analysed for PPRV antibodies. Also, 20 nasal swabs from domestic small ruminants showing respiratory signs were collected to check for presence of PPRV antigen. Competitive ELISA revealed highest proportions of anti-PPRV antibodies in domestic small ruminants around the Wildlife Park at Lahore (35%) as compared to Faisalabad (13%), with no existence of PPRV antibodies in tested serum of wild ruminants at these parks. Higher seropositivity was observed in females (25.6%) than in males (5.1%) and in goats (34.5%) compared to sheep (11.2%). The results of N-gene based RT-PCR highlight the absence of PPRV due to lack of current PPR outbreak in the region during study period. Even though grazing was not a significant risk factor, there is still a possibility of wildlife-livestock interactions for feed and water reservoirs, resulting in spillover of PPR to wildlife. Keeping in view the high seropositivity and risk of PPR, vaccination should be adopted to avoid circulation of PPRV among wild and domestic small ruminants (sheep and goats).

## 1. Introduction

In Pakistan, the livestock sector has a significant value in national gross domestic product (GDP) by contributing approximately 56.3% in the agriculture sector and almost 11.8% in Pakistan's GDP with Rs. 801.3 billion gross value of livestock. Total population of goat and sheep is 68.4 and 29.4 million with 44.6,000 tons of wool and 25.8,000 tons of hair, respectively, and collectively they are producing 671,000 tons of mutton annually [1]. It is assessed that peste des petits ruminants (PPR) cause annual losses of more than US$ 342 million through high morbidity and mortality rates, thus resulting in depletion of highly valuable genetic stock [2].

The causative agent of PPR is a virus (PPRV) in genus* Morbillivirus*, family Paramyxoviridae [3]. This virus has antigenic relationship to other viruses in the genus, such as measles virus (MV) that infects human; rinderpest virus (RPV), the infectious agent of the now eradicated cattle plague disease; canine distemper virus (CDV) that infects dogs; and porpoise, dolphin, and phocine-distemper viruses which cause infection in marine mammals [4]. PPRV was first discovered in the Ivory Coast in West Africa in 1942 [5] and was later found in Arabia [6], sub-Saharans countries [7], the Middle East [8], and southwest Asia [9]. PPR outbreaks have been reported in Pakistan, India, Nepal, Afghanistan, and Bangladesh [10]. In Pakistan, PPR has been recognized mainly in Punjab since 1991 [11] and infrequently recognized in different areas of the country during the subsequent years [12].

The clinical signs in diseased animals include pyrexia, nasoocular discharge, respiratory tract infection leading to pneumonia, ulcerations, and inflammation of the gastrointestinal tract leading to severe diarrhoea [13]. Following infection via respiratory tract, which is main portal of entry, PPRV replicates in oropharynx and mandibular lymph node. The incubation period of PPRV is about 3-4 days prior to onset of clinical disease [14, 15]. Viremia may develop within 2-3 days and via blood it spreads to other organs and tissues like spleen, lungs, bone marrow, and mucosa of gastrointestinal tract [16]. PPR is more pronounced in goats than in sheep [17], and mortality approaches to 100% when associated with other diseases, for example, goat pox [18]. PPRV infection is a transboundary animal disease (TAD), having high economic significance in domestic small ruminants [19]. Clinical signs of PPR have been reported in wildlife with deaths of Nubian ibex (*Capra ibex nubiana*), gazelles (*Gazella dorcas*), Laristan sheep (*Ovis orientalis laristanica*), bushbuck (*Tragelaphus scriptus*), impala (*Aepyceros melampus*), and gemsbok (*Oryx gazelle*). Similar to these, American white tailed deer (*Odocoileus virginianus*) has been experimentally infected with PPRV [20]. In 1987, an epidemic was reported in gazelles, ibex, and gemsbok of a zoo in the United Arab Emirates, the first outbreak in species other than domestic small ruminants according to International Office of Epizootics International animal health code [21]. There are often number of risk factors contributing to PPRV transmission among populations, including nomadic movement and exchange of animals in flocks [22]. Other factors such as sex, age, species, breed, and seasons have also been noticed [23].

In Pakistan, previous studies have focused on prevalence, isolation, and identification of PPRV from domestic small ruminants. However, there is less scientific data available about seroprevalence of PPRV antibodies in wild animals in the country. The aim of this study was to explore the occurrence of PPR in wild animals of different wildlife parks and in domesticated small ruminants living in close proximity in order to find out the possible role of disease transmission between wild animals and domestic small ruminants.

## 2. Materials and Methods

### 2.1. Samples Collection

Two wildlife parks (Gatwala Wildlife Park Faisalabad and Jallo Wildlife Park, Lahore, Pakistan) were targeted for serum collection not only from wild ruminants of these parks but also from domestic small ruminants. A total of 27 serum samples from wild ruminants and 238 serum samples from domestic sheep and goats were collected (Table 1). The sheep and goat herds were kept in vicinity of the selected wildlife parks for assessment of risk factors in transmission of PPRV. A total of 20 nasal swabs were collected from sheep and goats with respiratory signs (nasal discharge and cough) in concordance with PPR.

### 2.2. Serological Examination Using Competitive ELISA

Competitive ELISA was used on collected samples using standard kit, which was manufactured by CIRAD EMVT (Montpellier, France, distributed by BDSL with collaboration of flow laboratories). This immunological assay was conducted in Virology laboratory of National Veterinary Laboratories, Islamabad, following the recommended kit protocol. The value of threshold (cut-off value) was 50%; serum showing percentage inhibition (PI) <50% was considered as negative and >50% PI value showed positive samples indicating presence of PPRV antibodies.

### 2.3. Reverse-Transcriptase Polymerase Chain Reaction (RT-PCR)

A RT-PCR based on amplification of N protein gene using forward primer (N-PPR-f: 5′-TCT CGG AAA TCG CCT CAC AGA CTG-3′) and reverse primer (N-PPR-r: 5′-CCT CCTCCT GGT CCT CCA GAA TCT-3′) with probe sequence (N-PPR-p: FAM-5′-CGG CTG AGG CAC TCT TCA GGC TGC-3′BHQ1) was used for confirmation of PPRV by using commercial One-Step RT-PCR Kit (Qiagen) [24].

### 2.4. Statistical Analysis

Data from this cross-sectional study was distributed into two main groups of risk factors extrinsic risk factors (location and feeding pattern) and intrinsic risk factors (species, sex, and age). Associations between risk factors and outcome (being seropositive to PPRV) were analysed in a univariable analysis using chi-square test and one-way analysis of variance (ANOVA). A *p* value <0.05 was considered significant. All statistical analyses were performed using Minitab® version 17.1.0.

## 3. Results

**Figure 1:**
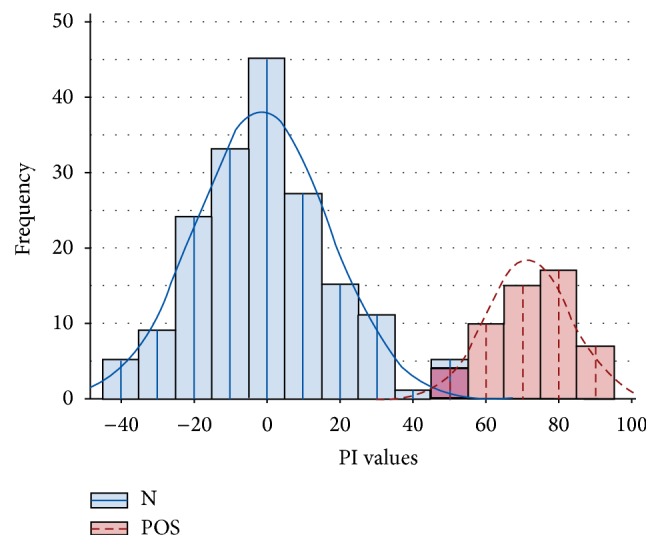
Frequency distribution of tested serum samples cELISA. (i) Positive serum PI values: 50 to 75. (ii) Strong positive serum PI values: 75 to 100. (iii) Negative serum PI values: less than 50.

Using cELISA 53 out of 238 serum samples were found seropositive for PPRV antibodies, corresponding to seroprevalence of 22.3% in sheep and goats. None of the wild ruminants were seropositive as shown in Table 2, and none of the nasal swabs out of 20 (collected from sheep and goats with observable respiratory clinical presentation) were found PPRV positive using RT-PCR. The distribution of PI values in the cELISA is shown in Figure 1.

### 3.1. Seropositivity Rate of PPRV in Targeted Samples

Samples were collected from sheep and goats in herds to determine the occurrence of antibodies against PPRV in vicinity of wild ruminant's habitat (wildlife park) in Lahore and Faisalabad as listed in Table 3.

### 3.2. Risk Factors Analysis

On the basis of statistical application, the results of univariates of different variables (locale, species, sex, age, and feeding pattern) are presented in Table 3.

### 3.3. Extrinsic Risk Factors (Location and Feeding Pattern)

The domestic small ruminants sampled in various villages of Lahore, surrounding Jallo Wildlife Park, showed significantly higher seropositivity (35%), as compared to various villages at vicinity of Gatwala Wildlife Park in Faisalabad (13.0%; *p* = 0.001). Feeding pattern was not found to be a significant risk factor for PPRV seropositivity.

### 3.4. Intrinsic Risk Factors (Species, Sex, and Age)

Goats were more prone to PPRV exposure with existence of 34.5% antibodies in tested animals as compared to sheep having 11.2% (*p* = 0.001). The strongest association for being seropositive for PPRV was sex (OR = 6.375), whereas significantly higher proportion of females (25.6%) than males (5.1%) were seropositive. There were no significant differences in seropositivity depending on age.

## 4. Discussion

The aims of the present study were to study presence of PPRV antibodies in wild and domestic small ruminants and to explore possible risk factors for seropositivity in domestic sheep and goats in the wildlife-livestock interface in villages surrounding wildlife parks in Lahore and Faisalabad, Pakistan.

In agreement with previous studies [25, 26], we found the PPRV seroprevalence to be higher in goats (34.5%) than sheep (11.2%). Differences in PPRV seropositivity depending on species, sex, age, season, and geographical location have previously been described [27]. In concordance to a previous study [28], significantly higher proportions of seropositive female sheep and goats compared to male animals were seen. This may be related to the physiological differences between female and male, where females reveal some degree of infection resulting from stress due to milk production and pregnancies. Due to significance of productivity potential, females maintained for a longer period of time as compared to males, thus increasing the likelihood for female animals to be exposed to PPRV over time. In contrast to current results [29] investigated that males were apparently more susceptible to PPRV infection than females.

The present study did not reveal significantly higher seroprevalence in animals older than 2 years of age (24.7%) compared to those younger than 2 years of age (13.5%). These findings are in contrast with our previous results that reported a liberal raise of seroprevalence with increasing age and related it to accumulation of recovered convalescent over time [30].

The reason of high occurrence of PPRV infection in Southern and Western part of Punjab is due to seasonal and climatic variation on forage availability, which influences the nomadic grazing pattern in Southern and northern part of Punjab [31]. In this study, there was no significant difference in seropositivity between stall feeding and grazing of animals, although this has been considered as a risk factor for acquiring infectious diseases [31]. One explanation could be that herds using stall feeding might have other management routines, such as purchase of animals [32], increasing the risk for PPR transmission.

In the present study, two cities (Lahore and Faisalabad) were targeted on basis of location of wildlife parks. According to geographical distribution of PPR, detection of antibodies against PPRV was significantly higher in Lahore (35.0%) compared to Faisalabad (13.0%). These results are in agreement with previous studies that detected antibodies against PPRV in small ruminants (sheep and goat) on the basis of geographical occurrence [33–38]. The higher seroprevalence in Lahore might be due to transmission of PPRV from India to Pakistan because of shared adjoining areas of Indo-Pak border.

The results of the present study revealed that there is no detection of PPRV antibodies in tested wild ruminants. Most likely, this was due to low number of sera samples contributed from wildlife and during this study there was no report of PPR outbreaks in the targeted area, but they have significant relationship in prevalence among wild and domestic ruminants according to previous studies. So, in future large data of sampling in wild species may show significant outcome regarding PPRV. Evidently, wildlife in Pakistan is susceptible to PPRV infection, as earlier studies showed the seroprevalence and detection of PPRV through clinical investigation and laboratory confirmation [30] and clinical picture of PPR in Sindh Ibex (*Capra aegagrus blythi*), confirmed by antigenic and serological analysis [39].

## 5. Conclusion

In this study, we show that geographical location, species, and sex are significant risk factors for seropositivity to PPRV in Punjab, Pakistan. Age and feeding pattern were not associated with significantly higher seropositivity, although they have been previously suggested as risk factors. All wildlife samples were seronegative, and further investigations are needed to show that wildlife-livestock interactions are increasing the risk of PPR transmission between wild and domestic species. The higher seroprevalence in Lahore compared to Faisalabad could suggest differences in, for example, herd management routines or wildlife-livestock interactions, contributing to increased circulation of PPRV in this area. Keeping in view the high risk of PPR infection, control strategies including vaccination should be adopted to avoid virus circulation at vicinity of wildlife parks to protect endangered species from PPRV infection.

## Figures and Tables

**Table 1:** Collection of serum samples from wild ruminants and domestic small ruminants which were kept in surrounding of Wildlife Park of Faisalabad and Lahore.

Sr. number	Animal type with binomial name	Lahore	Faisalabad
(1)	Sambar deer *Rusa unicolor*	9	0

(2)	Mouflon sheep *Ovis orientalis*	0	6

(3)	Hog deer *Axis porcinus*	0	3

(4)	Chinkara deer *Gazella bennettii*	2	2

(5)	Urial *Ovis orientalis vignei*	0	2

(6)	Blackbuck deer *Antilope cervicapra*	0	2

(7)	Nilgai (blue bull) *Boselaphus tragocamelus*	0	1

(8)	Domestic goats *Capra aegagrus hircus*	68	47

(9)	Domestic sheep *Ovis aries*	32	91

Total serum samples (wild ruminants)		27	
Total serum samples (sheep and goats)		238	

**Table 2:** Serological results of samples collected from targeted animal populations.

Competitive ELISA's results			
Samples	Wild ruminants	Goats	Sheep

Positive	0	39	14

Negative	27	74	111

Total	27	113	125

**Table 3:** Univariable analysis of host related determinants involved in antibodies based prevalence of PPRV.

Sr. number	Risk factors	Number of samples	Number of positive samples	% occurrence of PPRV	95% confidence interval	*p* value	OR
(1)	*Location*					0.001^*∗*^	3.59
	Lahore	100	35	35.0%	25.7; 44.3		
	Wagah Town	44	16	36.4%	22.2; 50.6		
	Jallo Basti	56	19	33.9%	21.5; 46.3		
	Faisalabad	138	18	13.0%	−3.4; 29.4		
	Chak Jumra	43	4	9.3%	0.6; 18.0		
	Chak 200 RB	30	5	16.7%	3.4; 30.0		
	Chak 199 RB	27	3	11.1%	−0.7; 22.9		
	Madina Town	18	6	33.3%	11.6; 55.0		
	UAF clinic	15	0	0.0%	−0.0; 1.0		
	G. M Abattoir	5	0	0.0%	−0.0; 1.0		

(2)	*Species*					0.001^*∗*^	4.18
	Goats	113	39	34.5%	25.3; 42.5		
	Sheep	125	14	11.2%	5.8; 16.8		

(3)	*Sex*					<0.001^*∗∗*^	6.38
	Female	199	51	25.6%	19.6; 25.7		
	Male	39	2	5.1%	−1.1; 12.0		

(4)	*Age*					0.15^NS^	0.47
	<2 years	52	07	13.5%	4.2; 22.8		
	≥2 years	186	46	24.7%	18.7; 30.9		

(5)	*Feeding pattern*					0.296^NS^	0.65
	Stall feeding	104	19	18.3%	10.8; 25.7		
	Grazing	134	34	25.4%	18.0; 32.7		

	Total	238	53	22.3%	16.9; 27.5		

^*∗∗*^High significance (*p* < 0.001); ^*∗*^significance (*p* < 0.05); NS: nonsignificance (*p* > 0.05).
